# Perspectives on Assessing the Flexibility of Hospitals for Crisis Mode Operations: Lessons From the COVID-19 Pandemic in the Netherlands

**DOI:** 10.1177/19375867231201633

**Published:** 2023-10-09

**Authors:** Liesbeth van Heel, Manuela Pretelt, Milee Herweijer, Clarine van Oel

**Affiliations:** 1 Department of Public Health, Erasmus University Medical Center (Erasmus MC), Rotterdam, the Netherlands; 2 Department of Architecture and the Built Environment, Delft University of Technology, the Netherlands; 3 Royal HaskoningDHV, Rotterdam, the Netherlands; 4 Wiegerinck, Arnhem, the Netherlands

**Keywords:** pandemic resilience, flexibility, robustness, staff adaptability, hospital design

## Abstract

**Background:**

The COVID-19 pandemic placed healthcare design at the heart of the crisis. Hospitals faced challenges such as rapidly increasing their intensive care unit capacity, enabling physical distancing measures, quickly converting to telehealth and telework practices, and above all, keeping patients and staff safe. Improving flexibility in hospital facility design and adaptability of hospital operations to function in “crisis mode” can be seen as ways of future-proofing for pandemics. In a design brief, flexibility is typically mentioned as an important target. Meanwhile, robustness of technical infrastructure is called for, and standardization at unit level with single-occupancy inpatient accommodation may be considered a way to enhance flexibility and adaptability in dealing with a surge in infectious patients.

**Aim:**

To future-proof facility design with pandemic preparedness and resilience in mind, this study evaluated what kinds of interventions were taken in Dutch hospital facilities and what perspectives need to be considered when hospitals operate in crisis mode.

**Methods:**

We have collected data from facility and estate professionals from 30 Dutch hospitals. Using a practice-based approach, in-depth interviewing helped uncover and compare successful operational strategies and design elements that provided the flexibility needed in the early stages of the recent crisis.

**Results:**

As we looked at existing facilities and alterations made to allow hospitals to operate during the COVID-19 pandemic, we discovered that staff availability and adaptability were deemed crucial.

**Conclusion:**

We add the perspective of staff as an essential factor to be considered when future-proofing hospital facility desigr crisis mode operation.

In early 2020, the COVID-19 pandemic overwhelmed the world. It turned out to be a multilayered crisis, hitting health systems in successive waves before reaching its current, more endemic stage. With large parts of the population in many countries vaccinated or with a better defense against severe illness after a prior infection, societies have opened up again since spring 2022. It is time to share findings from the first stages of the crisis and reflect on whether preparedness for future pandemics is now at an appropriate level.

Healthcare and healthcare buildings played an important role in facing the many challenges the COVID-19 pandemic brought to society. A sudden surge in intensive care unit (ICU) capacity was required during the first wave, and this required immediate facility changes (Stichler, 2021). The need to cohouse suspected COVID-19 patients and non-COVID-19 patients with different requirements enforced separate entrances and routes to keep staff and patients safe. These first few weeks forced infection prevention and control (IPC) experts and estate and facility managers to implement temporary or more permanent measures primarily based on their own expertise or guidance from national and international knowledge bodies, such as the World Health Organization. Since then, practitioners and scientists have collaborated globally to share insights and best practices on coping with the pandemic (Dietz et al., 2020; Hercules et al., 2020; World Health, 2020), and recently, reviews are also surfacing (Marmo et al., 2022).

The first wave of the pandemic underlined the importance of having a more flexible and versatile infrastructure to enable healthcare professionals to react and adapt quickly to suddenly emerging events while still providing all the services required in a hospital (Murphy, 2020). But with a rapid response in mind, the flexibility of buildings (and operations) has to be thought through at an early design stage (Memari et al., 2022). In a design brief, flexibility is typically mentioned as an important capacity for change. Some architects understand it in terms of the open building concept or future-proofing in healthcare building design (Capolongo et al., 2016; Karlsson et al., 2021). But it can also be associated with the concept of acuity adaptability in inpatient accommodation (Pati et al., 2008). Carthey et al. (2011) remark that reasons for hospitals’ need for change in capacity or capability of the built environment are often stated, but how well they do in practice is less well analyzed. In this sense, the recent COVID-19 crisis offers the opportunity for such an analysis.

A European collective of architects, consultants, and knowledge partners brought together case studies on pandemic resilience in a series of webinars and an online field guide. This “Relocate, Repurpose, Reorganize” project investigated how countries and healthcare organizations coped, focusing on four aspects: (1) supply chains, (2) space, (3) staff, and (4) systems (EuHPN, 2022). This field guide showcases not only what changes to consider when looking at spatial flexibility as the key to success for future-proof hospitals but also mentions awareness of higher capital investment. For hospital organizations, it has to do with optimizing the operational use of existing healthcare facilities, for example, by adding sectionable units, separating flows, plug-in units, temporary tented spaces, and using adjacent and repurposed buildings (EuHPN, 2022). New hospital design models were developed to create a pandemic setting with an “emergency hospital” within the hospital (Herweijer & Boonstra, 2020). This requires a modular design, with architectural and installation-technical design allowing for “crisis care” alongside “regular care,” adapting the care model when the need arises. Smart building technology helps separate flows, while “dormant” e-health solutions and hybrid working models for staff showed they could be implemented virtually overnight (Herweijer & Boonstra, 2020).

Pandemic preparedness thus far, and with history in mind, has focused on influenza type viruses overwhelming the world and health systems. In the absence of knowledge of the method of spread of a new pandemic disease, hospitals should prepare and take appropriate precautions to reduce airborne and contact transmission of a new virus (Gomersall et al., 2007). A prospective study from Australia stresses adequate staffing and staff training to strengthen their ability and confidence to work safely during a future pandemic (Dewar et al., 2014). In light of this pandemic preparedness and the ability to isolate patients, patient ward typology is also a building-related element to look at (EuHPN, 2022). In Europe and the Netherlands, the provision of 100% single-occupancy rooms is still fairly rare, while older facilities have to make do with a mix of single, double, or quadruple occupancy rooms. When renovating nowadays, hospitals introduce double occupancy rooms, expecting only one patient to be admitted but with the option of admitting another patient if more beds are suddenly needed. Other health systems are already more used to single-occupancy inpatient accommodation, seeing that this enhances flexibility in assigning rooms to infected or noninfected patients. It also improves patient safety by reducing the need for patient transfers with the associated risk of hospital-acquired infections (van der Schoor et al., 2022). In addition, when using additional testing on admitted patients and seeing that the actual virus shedding has stopped, isolation measures can be lifted on an individual basis without transferring a patient to another room (van Kampen et al., 2021). However, cohorted wards for the nursing of COVID-19 patients, even when single-patient room accommodation is available, have been advocated as well (Bardwell, 2022). From a staff perspective, a unit dedicated to COVID-19 care might provide nurses with a better feeling of control, as they are already in full personal protective equipment (PPE) and can enter a patient room straight away whenever the need arises. Both practices have advantages and disadvantages that resonate with the fundamental principle in preparing for a future pandemic that patient and staff safety must be ensured (Gomersall et al., 2007; Haanappel et al., 2023; van Dijk et al., 2021). Moreover, these opposite practices join the more general discourse about single- versus multi-bedded rooms that also seems to relate to cultural differences in dealing with infectious diseases (Zook & Sailer, 2022). So, more research on this particular subject seems to be needed (Bardwell, 2022).

Although health systems around the world adopted different coping strategies, overall, the Dutch situation compares with other European countries (Braithwaite, 2021). During the second wave, a prolonged use of (non-ICU) clinical capacity was seen for patients hospitalized with COVID-19 as registered by the *Landelijk Centrum Patiënten Spreiding* (LCPS; National Centre for Patient Distribution) and illustrated in Figure 1.

**Figure 1.**
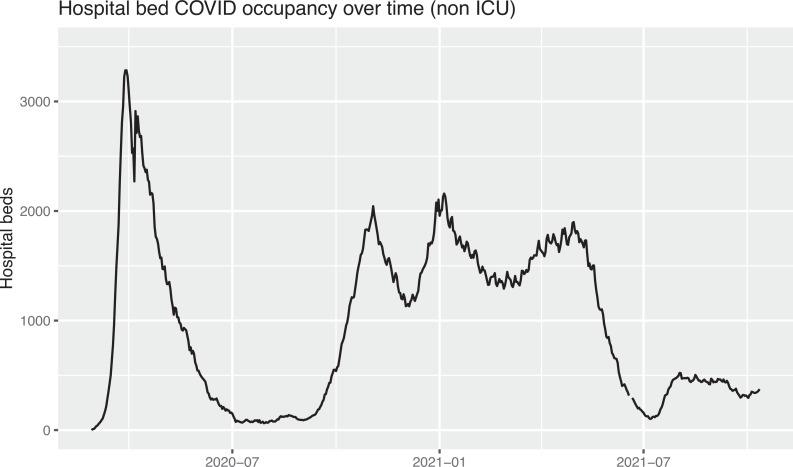
Dutch hospital beds dedicated to (non ICU) COVID-19 care during the first and second wave. *Source*: LCPS.

In their report, “Dancing with the Virus”, Dutch researchers identified five issues with the COVID-19 pandemic that needed to be balanced by governing bodies at the national, regional, and institutional levels: (1) availability of ICU capacity and PPE; (2) delayed care alongside coordinating and balancing COVID-19 and non-COVID-19 care; (3) the acute care chain (developing scenarios for a “code black”); (4) client representation (highlighting existing bottlenecks); and (5) burden on nurses (de Graaff et al., 2023). The aforementioned EuHPN field guide’s insights highlight, in a similar fashion to De Graaff et al., that enhancing preparedness for future pandemics requires focus on a combination of elements. But foremost, the pandemic showed the need to make rapid changes to facilities.

In the Netherlands, to prevent a “Code Black” that was anticipated by the end of March 2020 because of the increased need for ICU capacity, the policy response to the pandemic was initially framed as a very acute hospital crisis (Wallenburg et al., 2021). Based on this, we start with healthcare buildings in mind and explore where opportunities lie to design them prepared for another pandemic, and a potentially dramatic surge in capacity needs. Often the term “resilience” is used in preparing for dealing with and adapting to disruptions and disturbances after an immediate crisis (Nair & Howlett, 2016). It puts emphasis on effective action after a disturbance occurs and preparation for adjustments needed. Resilience in this sense might be understood as a combination of flexibility and robustness as well as adaptability. Robustness is then seen as the ability to avoid disproportionate collapse due to the initial damage and is associated with a more short-term and linear response than resilience (Nair & Howlett, 2016; Stochino et al., 2019).

The current study targets the physical and technical interventions taken by Dutch hospitals during the first and second waves of the pandemic, as well as additional services for staff. Using in-depth interviews with four estate and facility professionals who were identified through a survey into the chosen protective measures to evaluate these interventions, the perspectives of resilience as a combination of “flexibility” and “robustness” in future-proofing hospital design and operations were chosen. Therefore, the present study aims to gain insight from the kinds of interventions taken in Dutch hospital estates to inform and future-proof facility design or refurbishment with pandemic preparedness and resilience in mind.

## Dimensions of Resilience

Care delivery has rapidly changed over time following developments in biomedical science and technology. Consequently, hospitals’ architectural design needs to respond to the requirements of these technological developments and care delivery models. Pati et al. (2008) stated that architectural perspectives generally focus on expandability and convertibility to accommodate changing design needs while often not fully understanding their meaning from an end user’s viewpoint. They added flexibility to deal with different operational models or new circumstances at the unit level to the flexibility discourse at the time and referred to this kind of response to changing needs as adaptability. Resilience, here defined as the capacity to persist in disruptions and to survive and thrive during the building’s lifecycle, has become an additional requirement in hospital design at both the overall hospital and unit or ward level to comply with the evolution of medical knowledge and to future-proof for uncertain circumstances (Memari et al., 2022).

**Figure 2.**
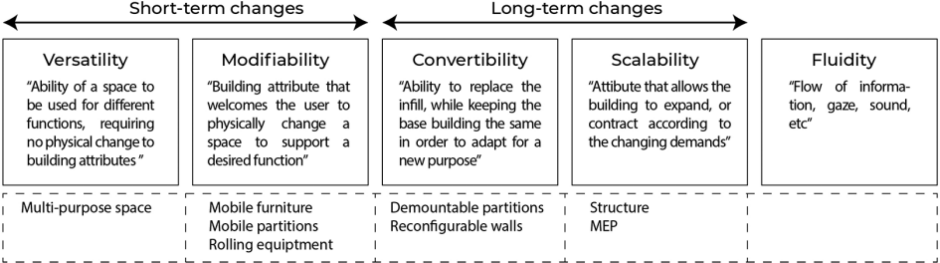
The five spatial dimensions of flexibility (after Monahan, 2002).

Flexibility in hospital design is a broad concept of accommodating changes, resulting in multiple perspectives being used by designers and practitioners in the field, as explained above, referring to the work done by Pati et al. (2008), Capolongo et al. (2016), and Karlsson et al. (2021). This study took its lead from Monahan (2002) in differentiating flexibility into five spatial dimensions as shown in Figure 2: versatility, modifiability, convertibility, scalability, and fluidity. Fluidity is understood to concern flows (of information, gazes and sound) in “here and now” and is less directly associated with space and other layers of the building and changes over time. Given the focus of this study being on facility design, we did not further address this dimension in the study. Versatility and modifiability relate to operational changes that can occur on a short-term basis, daily or weekly, and don’t require structural changes. In contrast, convertibility and scalability incorporate a more long-term perspective and are the elements of interest with expansion or reconfiguration in mind (Monahan, 2002).

Robustness can be understood as the ability to withstand or overcome adverse conditions while these conditions occur. This term is more associated with redundancy, continuity, and the prevention of errors in hospitals’ technical infrastructure and IT systems and operations (Tucker & Spear, 2006). Adaptability, meanwhile, is associated with designs that allow the implementation of changes over the life of a hospital (Pati et al., 2008). In our study, this term was also used to indicate the psychological and organizational resilience of staff: Design features offer the flexibility for staff to adapt (Pati et al., 2008).

## Method

### Setting

An online survey for real estate and facility managers in Dutch hospitals was developed. This specific group was targeted, assuming they would be most knowledgeable about the interventions taken in their facility. To maximize the response rate, great effort was taken to involve all relevant departments from all Dutch hospitals as included in the database of the National Institute for Public Health and the Environment (RIVM) with 68 health organizations identifying 117 hospital locations in 2019. In addition, the survey was advertised for 2 weeks on the website of *FMT Gezondheidszorg*, a trade magazine for the target population, to increase the rate of response. Also, the survey was distributed using the professional LinkedIn accounts of the authors.

The questionnaire was developed using Qualtrics software. It took approximately 10 min to complete and focused on hospital working practices (operations management) and building adaptations during the COVID-19 pandemic. The survey focused on how well hospitals felt they were prepared for a pandemic and what measures were taken in the various months after the start of the pandemic, not only with respect to the building and installations but also with employees in mind. Also, it tried to gather information on ward configuration and the numbers of beds and ICUs. The survey used can be found in the Supplementary Material. While developing the questionnaire and with the pandemic moving from the first wave into the second wave, the survey also asked whether COVID-19 and essential non-COVID-19 care were competing for resources. The online survey was distributed in March 2021. Informed consent was requested and given at the start of the survey. This study is part of a larger research project for which Institutional Review Board (IRB) permission has been sought and received.

In total, 38 responses were received, reflecting a net response rate of 56%. Among the respondents were six academic hospitals and 24 general hospitals. The distinction between general hospitals and academic hospitals was made based on their size and their role in the health system. Academic hospitals are large hospitals (>500 beds) where complex cases are referred. This was also the case with the first COVID-19 patient diagnosed in the Netherlands. They have a role as “last resort” for tertiary care patients, such as multitrauma patients, transplant patients, and so on. This implies part of their (ICU) capacity has to be reserved for emergency cases at all times, which means they played a less important role in the redistribution of COVID-19 patients across the country. Also, they have a large role in educating new doctors and nurses and enlarging Health Care Worker (HCW) teams providing care in clinics and wards. In academic hospitals, all doctors are salaried, while in general hospitals, medical specialists generally work in private practice, renting space and staff from the hospital.

There were eight respondents who filled out the questionnaire alongside a colleague from the same hospital organization. For building and technical interventions, a distinction was made between data from hospitals coming into use after 2010 (*n* = 6) and before 2010 (*n* = 24). In 2008, Dutch regulations on hospital capital investments changed, which might have led to different design priorities and tighter floor plans. As part of this deregulation, building notes were abolished, and hospitals gained more autonomy in making design decisions. Tighter floor plans, for example, by reducing corridor width, helped to reduce costs as hospitals became risk-bearing on these capital investments under the new regime. Likewise, the underutilization of capacities claimed and realized in the prior “risk-free” era could be reversed when the need for surge capacity was high. In our survey, the threshold was set at 2010 because it was argued that the consequences of this change in regulations would only become apparent from 2010 onward.

At the end of the survey, respondents were asked to reflect on what design changes they would recommend if they were to renew the current hospital or advise on a newly designed hospital. In addition, they were asked whether they would like to participate in an in-depth follow-up interview in May 2021. Interviews were made by LvH and CvO if the interviewee preferred Dutch; otherwise, the interviews were made by LvH and MP in English with the possibility of translating between English and Dutch. Three interviewees worked at academic hospitals; one represented a smaller general hospital. Since the spread of COVID-19 hit different regions at different times, we tried to obtain interviews from hospitals in these different regions. Two of the three academic hospitals are situated in the region, which was hit first and hardest, but they also had their specific role as a “last resort” within this area.

### Analysis

**Figure 3.**
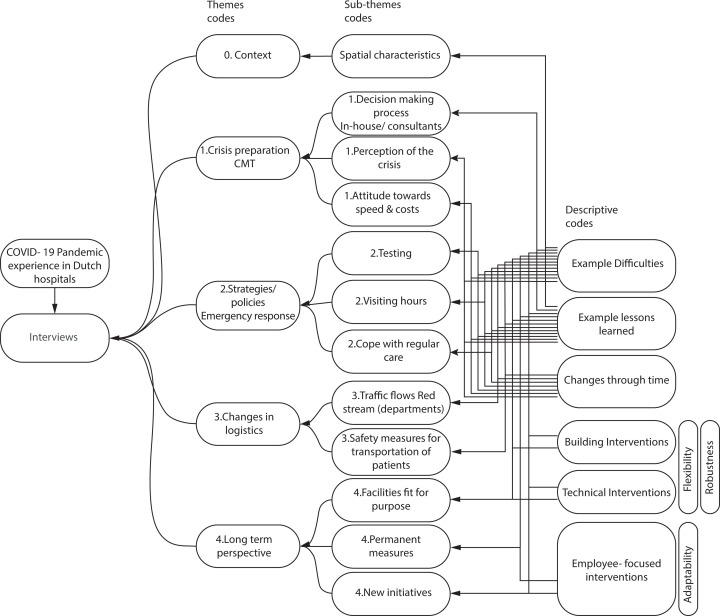
Code categorization and relations from the in-depth interviews.

The interviews were transcribed and analyzed using ATLAS.ti, using the code categorization and relations summarized in Figure 3. Flexibility and robustness of building and technical interventions and adaptability in staff-focused interventions were used as deductive codes; the remaining were inductive codes. Citations as used were translated from Dutch.

The outcomes of the survey and the follow-up interviews were first analyzed in a qualitative way, using visual counts. These counts have now also been statistically tested using SPSS.

## Results

**Figure 4.**
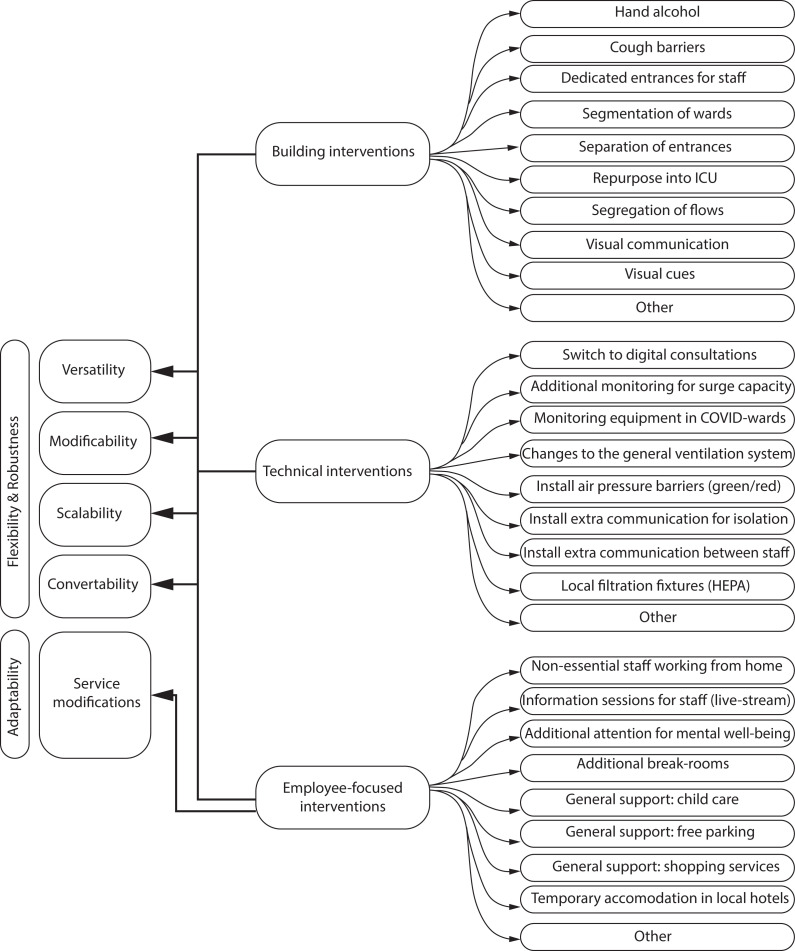
The building interventions, technical interventions and employeefocused interventions based on the framework of the online survey.

Following the framework used in the online survey, findings are presented in three subsections: building interventions, technical interventions, and employee-focused interventions as illustrated in Figure 4. However, while the aim of the study was to inform future facility design, the adaptability of HCW to deal with this more flexible use of the built and technical environment during the pandemic was added as an important notion. This adaptability is highlighted in a fourth subsection of the findings.

### Building-Related Interventions

The most enforced building intervention measure after the introduction of hand alcohol at hospital entrances was the segmentation of wards. The option to isolate infected patients within the building is essential to managing an infectious disease. Thus, dividing the building into “red” (COVID-19 positive), “green” (COVID-19 negative), and “amber” (COVID-19 suspected) zones should be considered in future developments.

“You have to be able to separate a part of your building for infected patients. Preferably a part without too much traffic around: an isolated part of the hospital” (Estate manager, academic hospital).

“As a major lesson from previous pandemics, it can be assumed that a future pandemic will also involve an influenza-type virus, resulting in infections with respiratory problems and the need for ventilation” (Estates manager, academic hospital).

***“As a major lesson from previous pandemics, it can be assumed that a future pandemic will also involve an influenza-type virus, resulting in infections with respiratory problems and the need for ventilation”***.

This statement about the continued risk of infectious diseases was acknowledged by all interviewees and related to growth in the world population and continued travel. Indeed, with a future pandemic in mind, some felt that hospital design should plan for surge capacity, not only in terms of ICU capacity but also in the nursing wards. The latter became particularly clear during the second and third pandemic waves, when the bottleneck was no longer the number of ICU beds. More was known about the virus and about ways to treat its symptoms at an earlier stage or in a less invasive way. Instead, the nursing wards, where many patients were treated with oxygen, became the focal point of concern. Future hospital design should consider the number of “ventilation beds” available with artificial respiration mechanisms like Optiflow ventilation and engineer the associated oxygen provision requirements. Medium-care wards could in this way help contain a future surge in demand as occurred in the COVID-19 pandemic crisis. Thus, hospitals are evaluating strategies to increase capacity through versatile and scalable spaces, like planning larger single-patient rooms that enable accommodation for two patients when necessary. As one of the interviewees mentioned,

In the wards, we have now double patient rooms and single-patient rooms, and the idea is that in the future we will use double rooms for one patient, and when we have a crisis, we can accommodate another person within that same room. (Estate manager, academic hospital)

Since academic hospitals have a different role in the nation’s health system, they need to keep spare capacity given their function as a last resort. Therefore, general hospitals played a larger role in redistributing the overflow of COVID-19 patients across the Netherlands. Especially during the first wave, regular care was scaled down and staff and spaces were repurposed to accommodate COVID-19 care. Given their features to ventilate and monitor patients at ICU level, holding/recovery areas or even operating theaters came into use as surge ICUs, causing elective surgical programs to be halted or severely reduced. Medical students and other HCW were engaged to help out.

Importantly, hospitals with older buildings mentioned fewer issues with repurposing spaces for ICU capacity, the separation of entrances, and the segregation of flows. The 2008 policy did not affect older buildings, in which the functional floor space of facilities was less limited and hospitals were designed less compact. This implied that buildings built before 2010 had more space to accommodate the surging demand.

“We had the luck that we have two big ICU units, and because of understaffing, one unit is almost always empty but could be reopened very quickly” (Estate manager, academic hospital).

Additionally, it was mentioned that multipurpose spaces are helpful in times of crisis. Hospitals found spaces like parking lots and other outdoor areas very convenient to install pop-up services or temporary structures directly connected to the building. Furthermore, inside the hospital, interviewees mentioned the need to store or install additional equipment, for example, in their laboratories.

There was a lot of equipment installed during the crisis, so they cleared out offices and filled them with the equipment. Another thing is that around our hospital we have space, mostly gardens and parking lots, and we claimed them when we had to put up tents. (Estate manager, academic hospital)

No significant differences were found between academic and general hospitals with respect to flexibility measures in relation to buildings.

### Technical Intervention

In the adoption of technical interventions, few differences were found between academic and general hospitals, with one exception. Although the numbers were small, nonparametric statistical testing showed that academic hospitals were quicker to introduce additional local ventilation fixtures (HEPA filters).

According to the survey results, the most common technical intervention was the transfer to digital consultations. This change is expected to become permanent, and the percentage of digital consultations might even increase.

“The video consultations have increased ten times during the crisis and are expected to continue after COVID-19” (Facilities and estate manager, general hospital).

Digital consultations have become a trend, and future hospital design needs to consider the impact of telehealth on the requirements that virtual sessions impose on the need for space and the robustness of infrastructure. As mentioned by one of the interviewees,

“We think about making small spaces that are only used for digital communication and that can be booked by various doctors to conduct their outpatient consultations,” and “The goal for the outpatient department is to reduce 50% of the physical visits to the hospital and replace them with video consulting” (Estate manager, academic hospital).

Switching between physical and video consulting also helped ensure physical distancing in outpatient waiting areas.

***Digital consultations have become a trend, and future hospital design needs to consider the impact of telehealth on the requirements that virtual sessions impose on the need for space and the robustness of infrastructure***.

During the interviews, it was discussed whether a reduction of 50% of in-person visits would be possible to achieve. Before the pandemic, reimbursement for digital consultations was a barrier to implementing telehealth. This has now been rectified and contributes to a more sustainable implementation of e-health solutions. However, given the fact that not all Dutch inhabitants have access to the necessary technological tools and their digital and health literacy may vary, virtual consultations could still prove to be too difficult for some patients.

The interpretation from the survey data was that general hospitals had to make more frequent and intrusive changes to technical installations compared to academic hospitals. Technical installations might be more advanced in academic hospitals or be related to design practices at the time of their construction. This was also elaborated on during the interviews. Some hospitals mentioned difficulties during the pandemic response with oxygen provision in the regular nursing wards because the building had insufficient overall capacity to safely increase the number of ventilated beds. This supports the concept of ensuring sufficient redundancy in technical infrastructure, resulting in robustness.

Another major issue concerns the ventilation system in use. Air pressure barriers were created at the beginning of the pandemic, and this was an adaptation mentioned by multiple hospitals. The survey results indicated that academic hospitals installed HEPA filtration units quicker than general hospitals. However, based on the interviews, it is not clear whether this is related to the complexity of care and the facility’s technical infrastructure or more generally to the age of the facility. When mild negative air pressure can be enforced in all single-occupancy patient rooms in a “COVID-19-ward,” the corridor can be considered a “safe zone,” where wearing PPE is not necessary. However, when the ventilation system is integrated into the whole building, it is not easy to tune the airflow hierarchy within or between departments or wards. “In the future, what is essential, is to create an isolation ward inside the ICU department instead of only creating isolation rooms” (Estate manager, academic hospital). From the interviews, it became clear that accommodation strategies should be convertible in terms of infrastructure and installations: “Ventilation adjustments can be made more permanently, so we don’t have to improvise things” (Estate manager, academic hospital).

Besides the technical infrastructure, interviewees explained that the existing IT infrastructure was able to support working from home and video consultations within a few days. The infrastructure had already been there, ready for the expected switch to more telehealth or telework, but there had not really been an incentive to use it beforehand. Likewise, one academic hospital could use the robustness of the patient monitoring infrastructure to upgrade the use of the single rooms from medium care to surge ICU. The interlude between the first and second waves was used to give some technical provisions a more permanent status. In these changes, nurses’ work processes and, for example, nurses’ wishes to have easier communication with a colleague inside the room or have a better overview of patients by adding a window in a previously solid door were accommodated as much as possible.

### Employee-Focused Interventions

Consistent with the national guidelines, the survey found that nonessential staff working from home was the most implemented employee-focused intervention. Staff working from home also applied for offices, where social distancing could otherwise not be upheld. One interviewee commented, “If social distancing limits the number of workspaces in the offices, working from home just needs to be enforced. We cannot expand the hospital to accommodate all staff at a 1.5-m distance” (estate manager, academic hospital). All hospitals set up communication lines to inform staff, both those working on site and those working from home, about day-to-day developments. Statistical testing of our survey results showed the number of measures targeting staff was significantly higher in academic hospitals compared to general hospitals. Compared to general hospitals, academic hospitals were seen to offer more free parking services (in collaboration with municipalities and in contrast to standard practice, which discourages staff from coming by car to inner city locations but to use public transport instead) and additional childcare services for HCW. Employee-focused interventions were found to be partly targeted at staff safety (e.g., free parking to reduce the risk with the use of public transport) and at staff morale (e.g., attention for mental well-being and other general support measures mentioned, such as shopping and child-minding services). Additional research would be needed to relate this finding to the size or location of hospitals, as urban density and HCW’s own social networks might play a role here.

With future facility design in mind, the size and technical infrastructure of break and meeting rooms need to be considered when facilitating staff social distancing and hybrid team meetings.

### Staff Adaptability

The robustness of these work processes was particularly challenged during the first wave, when medical students and other HCW were seconded to ICU wards to help out, for example, with turning patients, monitoring supplies, and performing “porter” services. Despite everyone’s willingness to collaborate and cross-traditional professional borders when hospitals went into “crisis mode,” it quickly became clear this could not be a structural situation. Short training courses can help prepare other HCW to assist with dedicated tasks and relieve workload, but they still need to be supervised by regularly trained all-round ICU nurses, adding to their particular burden of work. As one of the interviewees put it,

“The largest problem is not the hospital real estate; staff is the problem. So it’s not possible to increase hospital capacity by 50% and operate it with the same amount of staff. It is simply not possible” (Facilities and estate manager, general hospital).

So, in designing hospitals with resilience and staff adaptability in mind, staff well-being should receive a stronger emphasis.

The required adaptability of HCW, especially nurses, became an increasing burden during later waves. The facilities and estate manager of the general hospital also stated:

“Working with the Optiflow ventilation in a general ward generated additional work for the nurses”.

Additionally, doctors had to adapt to holding digital consultations with patients instead of seeing them in person. This should be considered when suggestions are made to significantly reduce outpatient facilities because of the further application of digital consultations.

## Discussion

This study showed that, in general, the Dutch hospital buildings were able to accommodate the first waves of the pandemic and the surge in patients. This is in line with findings in other countries (EuHPN, 2022). Although in the Netherlands, two large, standalone emergency facilities were planned and operational within weeks, the nonavailability of staff meant they never came into use. This echoes the findings in the United Kingdom regarding the so-called Nightingale Hospitals (Oliver, 2021). It underpins what was mentioned in all interviews: The availability of staff was the biggest bottleneck to dedicating more resources to COVID-19 care. The availability of a sufficient, sustainable frontline workforce in critical care areas, such as ICUs and emergency departments, was already identified in Australia as an essential measure for preparedness to accommodate the pressures of a future pandemic influenza in 2014 (Dewar et al., 2014). These critical care departments require staff with specific skill sets that need years of training. Although less skilled nurses and medical students could be freed up to assist, qualified nurses were not comfortable spreading their care over far more patients than they would normally care for. They had concerns about maintaining their standards of care.

***It underpins what was mentioned in all interviews: The availability of staff was the biggest bottleneck to dedicating more resources to COVID-19 care***.

Some hospitals had fully equipped ICU wards standing idle. With the redistribution of staff and the assistance of students, these could be quickly repurposed or recommissioned. Other hospitals had recently moved and could reopen an abandoned ward. The use of not yet opened or existing operating rooms as surge ICUs was also reported from Sweden and the United States, among other countries (EuHPN, 2022; Mittel et al., 2021). It would appear that hospitals that are in transition on their campuses have an advantage in repurposing empty spaces with high-tech infrastructure over more recently constructed hospitals, which already experience a tighter fit in floor plans relative to their production levels.

All these repurposing measures required, however, a vast reduction in “non-COVID-19 care,” as it became known during the first wave. During the second wave, the shortage of staff aggravated as hospitals found physical space for additional “ventilated” beds on the regular wards. Besides, the pressure to continue providing essential and urgent non-COVID-19 care became much higher (de Graaff et al., 2023). Patients needing more invasive therapies, such as Optiflow ventilation, also require additional monitoring by staff, whereas these kinds of monitoring systems (and skills) are usually not available outside the ICU. When technical infrastructure is not supporting this additional monitoring, direct sensory links (sight and hearing) to patient rooms need to be enhanced (Pati et al., 2008).

These findings resonate with earlier findings about acuity adaptable rooms, or universal rooms, that, from a design perspective, are seen as a way of future-proofing hospitals. The acuity-adaptable model for inpatient facilities, however, requires nurses who are multiskilled and, as such, cross-trained (or willing to be cross-trained) to address all levels of acuity. This is a challenge because nurses are typically trained to become specialists in one type of illness or injury. So, they have preferences for a certain type of care environment and level of acuity (Evans et al., 2008). However, it might reduce the cognitive load of staff working in a different environment while the hospital is in crisis mode when room design and ward layouts are standardized.

The practice of many Dutch hospitals to concentrate COVID-19 care in so-called cohorted wards (the whole ward deemed a red zone, with PPE worn by staff at all times and entrance to the ward restricted for visitors) is remarkable since a ward with the right air pressure regime and single-occupancy patient rooms offers the option. Such a ward configuration also make it possible to alter the “isolation regime” without moving the patient to another room. Indeed, several Dutch hospitals built after 2010 have opted for 100% single-occupancy rooms. But an aim to reduce “nonproductive” square meters, such as corridor width, to cut capital costs might hamper adequate space for a trolley with PPE supplies and the opportunity to don and dof based on an individual isolation regime. A possible explanation for the emerging preference for short-term, spatial flexibility measures such as the cohorted ward (not only in wards with a traditional configuration but also in combination with single-occupancy rooms) could be that nursing staff feel more in control as they have more overview and can check on their patients without losing time by putting on PPE (van Dijk et al.). It might also be related to a feeling of safety when wearing PPE at a time when a vaccine was not yet available. Although PPE had to be changed regularly during a shift in a cohorted ward, this might feel less time-consuming than donning (and doffing) for each individual patient contact. Indeed, this may suggest that spatial flexibility may interfere with the adaptive flexibility of staff: Nurses appear to feel more safe and at ease within the fixed setting and work practices of a cohorted ward, than in the more flexible setting with single-occupancy rooms, where they would need to switch between an isolation or nonisolation regime. This requires further research, especially as hospitals now have to care for patients carrying the COVID-19 virus but admitted for different procedures.

From the measures taken, such as setting up triage areas, introducing green and red zones and routes, social distancing, switching to digital consultations, and remote working, this study shows hospitals preferred to use flexibility measures that implicated the design of the building over technical interventions. All measures taken were instigated by the hospital’s own professionals, such as clinical, IPC, and real estate staff. There was simply no time to consult architects or engineering consultants. This suggests that, in planning and designing future facilities for pandemic resilience, the perspective of the hospital’s own stakeholders, like IPC experts, is essential (van Heel & van Oel, 2022).

### Limitations

Due to COVID-19-related circumstances at the time of this study, some limitations must be mentioned. Firstly, the in-depth follow-up interviews with real estate and facility managers at four different hospitals were conducted online. These circumstances also compelled us to waive interviews with frontline staff. It would have been interesting to also learn from the IPC experts of the various hospitals and include reflections from “crisis managers” themselves about the situation on the (cohorted) wards. However, we are grateful for the real estate and facility managers who could make time to talk to us. Secondly, the number of organizations participating in the in-depth interviews was quite low (four hospitals), and the number of academic hospitals versus general hospitals in our samples is not representative of the situation in the Netherlands. This can be seen as a limitation, as general hospitals had a different role in the redistribution of ICU-patients during the first wave. Finally, we also made use of the staff communications given by the academic hospital where the first author is employed; based on these communications, a case study was compiled that is referred to in the EuHPN (2022) report.

## Conclusions

The current study targets the physical and technical interventions taken by Dutch hospitals during the first and second waves of the pandemic. It also looked into additional services for staff as a means to gain insight into the kinds of interventions taken in Dutch hospitals that can inform future facility design. In doing so, this study used Monahan’s (2002) distinction between flexibility measures to future-proof that can be implemented without structural changes and therefore can be more rapidly deployed and measures requiring a more long-term perspective, such as in the case of hospital expansion or renovation. Robustness adds the perspective of operational continuity under stress (Tucker & Spear, 2006). (Acuity) adaptability indicated the resilience of staff (Evans et al., 2008; Pati et al., 2008). Although contextual factors influenced choices made, findings from this study show that flexibility and robustness in facility design and hospital operations are limited by the availability and adaptability of staff when hospitals are challenged to operate in crisis mode. However, when considering future-proofing, standardization of patient rooms and ward environments might be looked into, as this supports adaptability by reducing staff’s cognitive load when asked to flex between units for (crisis) operational reasons. Indeed, a combination of perspectives needs to be considered in assessing a hospital’s flexibility while operating in crisis mode and future-proofing its design for such an adverse eventuality. More research in this field, preferably with an interdisciplinary focus, is required.

***Indeed, a combination of perspectives needs to be considered in assessing a hospital’s flexibility while operating in crisis mode and future-proofing its design for such an adverse eventuality***.

## Implications for Practice

- Hospitals’ crisis mode operations should inevitably consider staff’s capacity to adapt to crucial work processes and environments as a limiting factor.
- There is a need to involve a hospital’s internal stakeholders, like estate and facility managers, but also IPC experts in future-proofing hospital facility design. They are part of the multidisciplinary team relied upon to create a first response to unforeseen adverse situations.
- Single-occupancy patient rooms, with an appropriate air pressure and ventilation regime, should be considered as a way to isolate patients on a “need to isolate” basis. This reduces transfers and enables staff to adapt to COVID-19 and non-COVID-19 care within the care environment they are accustomed to.
- Standardization of patient rooms and ward environments should be looked into, as this supports adaptability by reducing staff’s cognitive load when asked to flex between units for (crisis) operational reasons.
- Further digitalization of consultations not only requires suitable infrastructure in a hospital’s design but should also consider health and digital literacy at the patient’s end of the conversation.

## Supplemental Material

Supplemental Material, sj-pdf-1-her-10.1177_19375867231201633 - Perspectives on Assessing the Flexibility of Hospitals for Crisis Mode Operations: Lessons From the COVID-19 Pandemic in the Netherlands Click here for additional data file. Supplemental Material, sj-pdf-1-her-10.1177_19375867231201633 for Perspectives on Assessing the Flexibility of Hospitals for Crisis Mode Operations: Lessons From the COVID-19 Pandemic in the Netherlands by Liesbeth van Heel, Manuela Pretelt, Milee Herweijer and Clarine van Oel in HERD: Health Environments Research & Design Journal
