# Supplementary material for: Perspectives on Assessing the Flexibility of Hospitals for Crisis Mode Operations: Lessons From the COVID-19 Pandemic in the Netherlands
Source: HERD. 2023 Oct 9;17(1):34–48. doi: 10.1177/19375867231201633 (PMC10704891; doi:10.1177/19375867231201633)
Supplement: Supplemental Material, sj-pdf-1-her-10.1177_19375867231201633 - Perspectives on Assessing the Flexibility of Hospitals for Crisis Mode Operations: Lessons From the COVID-19 Pandemic in the Netherlands [file sj-pdf-1-her-10.1177_19375867231201633.pdf]

Supplemental material: Appendix 1

Survey "Your hospital during COVID-19"

What makes this project special is that we would like to learn how we can futureproof the design of hospital buildings to pandemics like COVID-19.

Would you like to participate in this survey and start the online questionnaire?

If you indicate below that you wish to participate in the study, you consent to the collection, storage, and inspection of the data you provide.

- Yes, I would like to participate in the study
- No, I would rather not participate in the study

Q1 What is your position in the healthcare organization?

- Real estate manager
- Facility/Real Estate advisor
- Facility/Real Facility manager
- Estate director
- Logistics manager
- Other, namely

Q2 Please fill out for how long have you worked in your current position? (# of years)

Q3 What is the highest degree or level of education that you have completed?

- Vocational college
- BSc
- MA/MSc
- Other, namely

This questionnaire is focused on the preparedness of the hospital building during the COVID-19 pandemic. The first section is about general characteristics of the facilities, then the survey will center on the operations and adaptations made to the buildings to respond to the outbreak. Finally, the last questions are about the future of the hospital after the pandemic experience. It is very valuable that you

answer the whole questionnaire although you may not have all the specific information. If there is someone else in your organization who can answer further questions about COVID-19 measures taken in your hospital, please fill out your colleague’s contact details at the end of the questionnaire.

Q4 Please fill out the zip code of the hospital you work for. If the hospital has more than one location, fill out the zipcode of the hospital's main location you are responsible for. (4 digits zip code)

Q4.A If your hospital had more than 1 location, did you have a location that was 100% dedicated to non-COVID care (yes/no/other)?

Note: If your hospital has assigned locations only for COVID-19 patients: please answer the questions of the survey regarding the hospital's (main) location assigned to COVID-19 care.

Q5 Please state the total number of beds in the hospital prior to the pandemic (number of beds)

Q6 Please state the number of intensive care places prior to the pandemic (number of ICU-beds)

Q7 Please indicate the percentage of single patient rooms in the inpatient wards prior to the pandemic (%)

Q8 Did your hospital use cohort nursing for COVID-19 patients (yes/no)?

Q9 Did your hospital collaborate with local hotels and/or other care organizations to free up hospital beds for COVID-19 patients?

- Yes, during the first wave (Feb 2020 - July 2020)
- Yes, during the second wave (Aug 2020 - Feb 2021)
- Yes, in both waves
- No
- I don't know

Q10 How well prepared do you consider your hospital was for the different stages of the COVID-19 pandemic ? Mark a number on the scale between 0 to 10. Considering 0 as not at all prepared and 10 as very well prepared (give mark for each period).

- For the first wave
- For the second wave
- For the British variant (third wave)

Q11 Could you indicate if and in which month the following **physical interventions** were introduced in your hospital? Please choose the year and fill out the number of the month in which the measure was implemented. If the measure was not introduced, please fill out the number 0.

Physical intervention measure	Year			Month
	2020	2021	I don't know	0, 1 to 12
Put up visual cues (e.g. limiting amount of people in elevators, routes marked on floor)				
Provision of hand-alcohol at entrances				
Install cough-barriers between patients and staff at desks				
Enhance visual communication between staff (glass in doors, plastic barriers)				
Repurpose spaces into ICU-capacity				
Separation of entrances for infected and non-infected patients				
Segmentation of wards with infected and non-infected patients				
Segregation of ‘red’ (potentially infected) and ‘green’ (triaged, non-infected) flows (patients, visitors and staff) throughout the hospital				
Dedicated entrances for staff				
Other, namely				

Q12 Could you indicate if and in which month the following **technical interventions** were introduced in your hospital? Please choose the year and fill out the number of the month in which the measure was implemented. If the measure was not introduced, please fill out the number 0.

Technical intervention measure	Year			Month
	2020	2021	I don't know	0, 1 to 12
(Partial) switch to digital consultations				
Advanced monitoring (equipment) in COVID-wards				

Additional monitoring for surge ICU-capacity				
Install air-pressure barriers between ‘red’ and ‘green’ zones				
Install extra communication systems (for patient in isolation)				
Install extra communication systems (between staff)				
Changes to the general ventilation systems				
Local filtration fixtures (HEPA)				
Other, namely				

Q13 Could you indicate if and in which month the following **services for staff** were introduced in your hospital? Please choose the year and fill out the number of the month in which the measure was implemented. If the measure was not introduced, please fill out the number 0.

Services for Staff	Year			Month
	2020	2021	I don't know	0, 1 to 12
(Partial) switch to digital consultations				
Advanced monitoring (equipment) in COVID-wards				
Additional monitoring for surge ICU-capacity				
Install air-pressure barriers between ‘red’ and ‘green’ zones				
Install extra communication systems (for patient in isolation)				
Install extra communication systems (between staff)				
Changes to the general ventilation systems				
Local filtration fixtures (HEPA)				
Other, namely				

Q14 Are you planning to have a refurbishment or renovation of (part) of your hospital building (yes/no)?

Q14.A Are you already including COVID-19 measures in your facilities to futureproof hospital building to (yes/no)?

Q15 Assuming there will be a renovation in the coming years, which interventions related to COVID-19 would you recommend to be included in the design?

Q16 Do you have a floor plan of the hospital that you could share with us for this research project? Could you please email the file to email of student.
It would be useful if the floorplan contains the departments most involved in delivering the COVID-care, such as: emergency department, ICU, and nursing wards (yes/no).

Q 17 Would you like to participate in the prize draw (yes/no)?

Q18 Would you like to receive a summary of the results of this study (yes/no)?

Q19 We are planning to do additional interviews to better understand the outcomes of the survey. Are you willing to participate in a follow-up interview? You do not have to decide now whether you really participate, you will only be asked if we can approach you with a request. You can always refuse.

- Yes, you can use my data to contact me again with a request to participate in an interview to complement the research.
- No, I do not want to be asked again to participate in this research project.

Q20 Would you please leave your contact details below (name, email)?

Q21 You may feel that we should (also) contact a colleague about this research or the follow-up interview. If so, could you please leave their contact details here as well (name, email)?

Q22 Is there anything you would like to advise, mention or comment?
